# Distinct Roles of NMDA and AMPA Receptors in the Pathogenesis of Hyperbaric Oxygen-Induced Seizures

**DOI:** 10.3390/biomedicines14081807

**Published:** 2026-08-11

**Authors:** Olga Alekseeva, Karim Zaripov, Dmitry Vasilev, Ivan Demchenko

**Affiliations:** Sechenov Institute of Evolutionary Physiology and Biochemistry of the Russian Academy of Sciences, 44, Toreza Prospekt, Saint Petersburg 194223, Russia; osa72@inbox.ru (O.A.); zaripovkarim@gmail.com (K.Z.); dvasilyev@bk.ru (D.V.)

**Keywords:** hyperbaric oxygen, CNS oxygen toxicity, NMDA, AMPA, memantine, perampanel

## Abstract

**Background:** The mechanisms underlying central nervous system oxygen toxicity (CNS-OT), which manifests as generalized seizures, remain poorly understood, thereby limiting the utility of hyperbaric oxygen therapy and deep-sea diving. This study investigates the distinct contributions of ionotropic N-methyl-D-aspartate (NMDA) and alpha-amino-3-hydroxy-5-methyl-4-isoxazolepropionic acid (AMPA) glutamate receptors to hyperoxic seizure initiation and hippocampal neurodegeneration. **Method:** Male Wistar rats were assigned to seizure-monitoring groups (*n* = 86) exposed to 100% O_2_ at 6 ATA until seizure onset: saline control; memantine (1, 5, 25 mg/kg); perampanel (0.2, 1, 5 mg/kg); memantine + perampanel combination (5 + 1 mg/kg); and methionine sulfoximine controls (50, 100 mg/kg). For morphological analysis (NeuN immunohistochemistry, 48 h post-exposure), separate groups (*n* = 8 per group) were exposed to HBO_2_ for a fixed 30 min duration, alongside a normobaric room-air control (*n* = 8). Seizure latency and progression (Racine scale) were analyzed via one-way ANOVA and Tukey’s test. **Results:** Paradoxically, NMDA receptor blockade with memantine completely failed to protect animals, significantly accelerating seizure onset (*p* < 0.05). Conversely, non-competitive AMPA inhibition with perampanel (1, 5 mg/kg) exerted a powerful, dose-dependent anticonvulsant effect and prevented hippocampal CA1/CA3 cell death. Perampanel co-administration completely mitigated the proconvulsant acceleration induced by memantine, demonstrating that AMPA receptor activation, rather than NMDA signaling, serves as the true primary upstream trigger for hyperoxic convulsions. **Conclusion:** Our findings sharply contrast with the established priority of NMDA glutamate receptors in epileptogenesis, revealing a novel neurochemical mechanism under extreme hyperoxia. We identify fast-acting AMPA receptors as the definitive principal molecular targets for mitigating CNS oxygen toxicity.

## 1. Introduction

Pure oxygen (≈100%) at pressures exceeding 1 atmosphere absolute (ATA) is widely utilized for therapeutic purposes in hyperbaric oxygen therapy (HBOT) and as a breathing medium in deep-sea diving. While hyperbaric oxygen (HBO_2_) is widely recognized as a safe and effective clinical modality for treating conditions like carbon monoxide poisoning or chronic wounds, its physiological effects are strictly dependent on pressure and duration. Clinical protocols typically utilize lower pressures (usually 1.5 to 2.5 ATA) for limited periods (60 to 90 min). In contrast, exposure to higher oxygen pressures (typically 3–6 ATA) is well-documented to induce central nervous system oxygen toxicity (CNS-OT). Therefore, high-pressure HBO_2_ exposure is explicitly employed in biomedical research not as a therapy but as a robust and reproducible experimental model to investigate the mechanisms of oxygen-induced seizures and evaluate potential neuroprotective interventions.

Moreover, while HBO_2_ is generally considered safe within established clinical limits, exposure to pressures above 2 ATA for prolonged periods (tens of minutes) significantly increases the risk of CNS-OT [1,2]. This dangerous threshold means that the potential for CNS-OT remains a major limiting factor for both hyperbaric oxygen therapy and deep-sea diving operations, where accidental exposure to elevated pressures can trigger severe convulsions. This form of neurotoxicity typically manifests as generalized motor seizures, characterized by involuntary tonic and clonic muscle contractions that are nearly identical to the generalized tonic–clonic seizures observed in epilepsy patients [3,4]. These HBO_2_-induced convulsions are accompanied by paroxysmal electrical activity in the brain, manifesting as epileptiform spikes on the electroencephalogram (EEG). Clinically, such seizures are often preceded by prodromal symptoms, including visual and auditory impairments, nausea, and focal facial twitching, all stemming from acute neuronal hyperexcitability [5].

Despite the clear clinical and electrophysiological similarities between CNS-OT and epilepsy, the precise molecular triggers of hyperoxic convulsions remain poorly understood. There is a growing body of evidence that reactive oxygen and nitrogen species (RONS), including primary molecules such as the superoxide anion (O_2_^•–^) and nitric oxide (NO), are key mediators of HBO_2_-induced seizures [6,7]. These species, along with secondary derivatives including the hydroxyl radical (•OH), hydrogen peroxide (H_2_O_2_), and peroxynitrite (ONOO–), drive oxidative stress, leading to neuronal damage and seizure initiation. Neuronal membranes are particularly vulnerable to RONS due to high concentrations of polyunsaturated fatty acids and membrane-bound proteins within various receptors and ion channels. RONS-induced reactions, such as lipid peroxidation, protein nitration, and S-nitrosylation, can alter membrane properties and disrupt the function of embedded proteins—particularly ion channels, neurotransmitter transporters, and enzymes—leading to cellular dysfunction and enhanced excitatory processes [8,9]. Experimental studies have shown that HBO_2_-derived RONS enhance excitability in neuronal networks [10] and reduce membrane ionic conductance, thereby increasing the firing rate of central neurons [11].

Since RONS have a very short biological half-life, their targets are located in close proximity to the sources of these redox molecules. One potential target for initiating oxygen convulsions is GABAergic neurotransmission. Hyperbaric oxygen—or more specifically, RONS—induces neurotoxicity by inhibiting glutamic acid decarboxylase (GAD) activity, which is crucial for neurotransmitter balance. This occurs via S-nitrosylation, specifically where nitric oxide binds to cysteine thiol residues on GAD65, as evidenced by a 50% decrease in GAD activity [12]. Reduced GAD activity diminishes GABA production, leading to insufficient inhibition in the central nervous system; this disrupts the balance between glutamatergic and GABAergic synaptic transmission and triggers hyperexcitability and seizures [13].

Other targets for RONS reside within glutamatergic neurotransmission, which involves a cycle of glutamate release from presynaptic terminals, activation of postsynaptic ionotropic/metabotropic receptors, and clearance via transporters into presynaptic neurons and astrocytes. In astrocytes, glutamate is converted to glutamine. Virtually all components of glutamatergic neurotransmission are potential targets for RONS, as they encompass proteins susceptible to post-translational modifications, most notably S-nitrosylation and the oxidation of critical thiol groups [9,14]. Ionotropic glutamate receptors, specifically the NMDA and AMPA subtypes, are fundamental components of the glutamatergic system that facilitate rapid excitatory synaptic transmission. Within the framework of epileptogenesis, the redox-mediated modulation of these receptors is hypothesized to play a pivotal role in disrupting neuronal electrogenesis and the propagation of electrical signals [15]. These neurophysiological aberrations ultimately manifest as synchronized epileptiform discharges on EEG recordings and the presentation of clinical motor seizures [16].

Historically, NMDA receptors have been the most extensively studied targets in seizure research. They are primarily associated with epileptogenesis due to their fundamental role in calcium-mediated neurotoxicity. In vitro studies have demonstrated that these receptors amplify excitatory signaling via intracellular calcium influx, which triggers neuronal hyperexcitability and facilitates seizure progression [17]. In various epilepsy models, NMDA receptor antagonists have been shown to prevent or attenuate epileptogenic processes; however, their anticonvulsant efficacy often varies, depending on their subunit specificity and the particular stage of seizure development [18,19].

AMPA receptors mediate rapid excitatory neurotransmission [20]. These receptors contain an ion-selective channel that remains closed in the absence of glutamate. Upon glutamate binding, AMPA receptor channels open, allowing cations to cross the postsynaptic membrane and induce a transient depolarization known as an excitatory postsynaptic potential (EPSP). Although AMPA receptors are permeable to sodium, potassium, and occasionally calcium, sodium serves as the primary carrier of the depolarizing current at the resting membrane potential. The resulting EPSPs trigger action potentials in the postsynaptic neuron, thereby completing synaptic signal transmission [16].

The involvement of NMDA receptors in the development of HBO_2_-induced seizures has been explored in only a limited number of studies. In vitro experiments have shown that pre-blockade with the NMDA receptor antagonist MK-801 protects cultured neurons from damage induced by HBO_2_ at 6 ATA [21]. Specifically, NMDA receptors containing the GluN2A subunit exhibit enhanced responses at an oxygen pressure of 6.3 ATA, whereas GluN1/GluN2B combinations appear insensitive to this level of hyperoxia [22]. Further investigations using the Zn^2+^ chelator TPEN suggested that the increased current through GluN1/GluN2A receptors is linked to the elimination of high-affinity, voltage-independent inhibition by Zn^2+^. Based on these findings, it has been proposed that HBO_2_ acts by displacing Zn^2+^ from its specific binding site on the N-terminal domain of the GluN2A subunit, thereby promoting hyperexcitability [23]. In contrast, in vivo studies regarding NMDA receptor function under extreme hyperoxia are sparse and yield conflicting results. In awake rats exposed to 6 ATA of oxygen, low doses of MK-801 were found to paradoxically accelerate the onset of motor seizures, while a higher dose of 8 mg/kg had no effect on seizure latency [24]. Conversely, in anesthetized rats under 5 ATA oxygen, a high dose of MK-801 (12 mg/kg) reportedly attenuated the development of epileptiform activity on the EEG [25].

To date, the involvement of AMPA receptors in hyperbaric oxygen-induced seizures has not been investigated. The introduction of perampanel, the first-in-class, selective, noncompetitive AMPA receptor antagonist, offers a unique pharmacological approach to inhibiting fast excitatory neurotransmission. Its successful clinical utility in the management of drug-resistant focal seizures, as well as primary generalized tonic–clonic seizures, is well established [26,27].

Both AMPA and NMDA receptors are essential for consolidating neurotransmitter signaling and maintaining membrane potential, facilitating both basal synaptic processes and complex cognitive functions. In the context of epilepsy pathogenesis, AMPA receptors are primarily responsible for mediating fast excitatory responses, whereas NMDA receptors are crucial for downstream signaling and synaptic plasticity [28,29]. However, despite extensive research on glutamate toxicity, the precise roles and relative contributions of NMDA versus AMPA receptor pathways in the acute phase of HBO_2_-induced seizures are still not fully understood. Based on these distinct physiological profiles, we hypothesized that AMPA receptors play a more critical, immediate role than NMDA receptors in the initiation and propagation of hyperoxic seizure activity.

To resolve this issue, the primary goal of this study was to elucidate the neurochemical mechanisms underlying the initiation of CNS-OT seizures by differentiating the specific contributions of NMDA and AMPA receptor pathways. To achieve this goal, we focused on the following specific objectives:

1. To evaluate and compare the individual, dose-dependent anticonvulsant efficacy of the non-competitive NMDA receptor antagonist (memantine) and the selective AMPA receptor antagonist (perampanel) in a well-established rat model of hyperbaric oxygen toxicity.
2. To investigate the potential synergistic or additive protective effects of combined NMDA and AMPA receptor blockade using concurrent memantine and perampanel administration.
3. To determine the morphological status of hippocampal neurons following high-pressure HBO_2_ exposure and to evaluate the specific protective impacts of two glutamate receptor antagonists, memantine and perampanel.

## 2. Materials and Methods

### 2.1. Animals and Chemicals

Adult (3-month-old) male Wistar rats weighing 220–250 g were obtained from Rappolovo Laboratory Animal Facility, Leningrad Oblast, Russia. Upon arrival, rats were housed in groups (4 rats per cage) and maintained in a temperature-controlled room (21–23 °C) on a 12 h light/dark cycle. Rats had free access to food and water and were acclimatized for at least 5 days before experiments. Rats in the control and experimental groups were mixed from different litters to exclude the influence of genetic factors. All experimental procedures were reviewed and approved by the Bioethics Committee of Sechenov Institute of Evolutionary Physiology and Biochemistry of the Russian Academy of Sciences (approval protocol No. 1-3/2025, dated 30 January 2025). All procedures were performed in accordance with the institutional guidelines for animal care and use and complied with Directive 2010/63/EU of the European Parliament and of the Council on the protection of animals used for scientific purposes.

Chemicals (memantine, methionine sulfoximine—MSO) were purchased from Sigma Chemical Co. (St. Louis, MO, USA); perampanel (sold under the trade name Fycompa) was obtained from Eisai Co., Tokyo, Japan.

### 2.2. Hyperbaric Oxygen Seizure Model

The detailed model of hyperbaric oxygen-induced seizures, involving the exposure of rats in a pressure chamber at 4–6 ATA, is widely utilized and thoroughly described in numerous studies [3,4,25]; therefore, we provide only a brief overview here.

Following drug administration, animals were placed in a 100 L hyperbaric chamber for a single HBO_2_ exposure session, with four rats exposed simultaneously. The chamber was equipped with dedicated inlets and outlets for continuous flow ventilation, as well as for controlled compression and decompression processes. To monitor the animals, two transparent viewports were integrated into the chamber setup, allowing for internal illumination and direct observation of animal behavior. Additionally, the setup featured a chemical carbon dioxide (CO_2_) scrubbing system to prevent gas accumulation and a multichannel hermetic feedthrough for the real-time registration of physiological parameters and environmental temperature monitoring. All key experimental parameters, including compression/decompression rates, oxygen concentration, and temperature, were adjusted and monitored via an external control console located outside the chamber. The HBO_2_ exposure protocol consisted of a single session. Compression was performed using 100% oxygen at a constant rate of 1 ATA/min until reaching the treatment pressure of 6 ATA. The total exposure duration at peak pressure was maintained until the onset of generalized seizures (or for a maximum period of 90 min). During the session, the temperature inside the chamber was maintained within 23–25 °C, relative humidity was kept at approximately 60%, and the CO_2_ content did not exceed 0.05%. Following the exposure, a controlled decompression procedure was carried out for a total duration of 8 min. During HBO_2_ exposure, continuous video recording of the animal’s behavior was performed. Seizure latency (time from reaching stable pressure (6 ATA) to the first generalized seizure) and seizure severity (using the modified Racine scale) were monitored by two observers blinded to the treatment groups. Hyperbaric exposure continued until generalized tonic–clonic seizures appeared in all rats. In the absence of generalized motor seizures, the seizure latency was taken as 90 min. Motor seizure progression was assessed via continuous video monitoring and staged according to a modified Racine scale [30]. Seizure severity was categorized into four distinct stages: (1) orofacial movements; (2) head nodding; (3) forelimb clonus; and (4) rearing and falling with tonic–clonic activity. For analytical purposes, stage 4 was defined as a generalized convulsive seizure.

### 2.3. Study Design and Animal Groups

Prior to HBO_2_ exposure, animals received an intraperitoneal injection of one of the following drugs: memantine, perampanel, methionine sulfoximine (MSO), or saline. The doses of these chemicals were chosen based on established literature data demonstrating their neuroprotective or metabolic efficacy in rodent models of epilepsy [18,20]. Sterile saline (0.9% NaCl) was administered in an equivalent volume to the control group to account for the potential physiological effects of the injection procedure itself. Memantine was administered 1 h before exposure, dissolved in distilled water. Perampanel was administered 30 min before exposure, dissolved in a mixture of distilled water, dimethyl sulfoxide (DMSO), and polyethylene glycol-200 (PEG-200) in a 1:1:1 ratio. MSO was administered 6 h before exposure at doses of 50 and 100 mg/kg, dissolved in distilled water. All drugs were administered in a volume of 1.0 mL per rat.

Animals were randomly assigned to the initial treatment groups to monitor seizure onset: saline control group exposed to HBO_2_ at 6 ATA (vehicle, *n* = 14); MSO pre-treated groups (50 or 100 mg/kg, *n* = 8 per group) along with a separate saline control group exposed to HBO_2_ at 5 ATA (vehicle, *n* = 8); memantine pre-treated (1, 5, or 25 mg/kg, *n* = 8 per group); perampanel pre-treated (0.2, 1, or 5 mg/kg, *n* = 8 per group); and a memantine + perampanel combination group (5 + 1 mg/kg, *n* = 8). All injections were administered intraperitoneally (i.p.).

For subsequent morphological analysis, four separate, additional groups of rats (*n* = 8 per group) were used and exposed to HBO_2_ at 6 ATA: HBO_2_ control, memantine (5 mg/kg), perampanel (1 mg/kg), and the combination group (5 + 1 mg/kg). Animals from all four experimental groups were exposed to HBO_2_ for a fixed duration of 30 min. This standardized exposure time corresponds to the lower threshold for seizure onset in control animals, which was previously determined during our evaluation of behavioral seizure progression stages. A separate group of rats (*n* = 8) breathing room air under normobaric conditions served as the morphological normobaric control. A total of 134 rats were used in this study.

### 2.4. Morphological and Immunohistochemical Analysis of Hippocampal Neurons

Forty-eight hours following HBO_2_ exposure, the rats were anesthetized with isoflurane (Laboratorios Karizoo, Barcelona, Spain) and euthanized by decapitation. The brains were rapidly harvested for further examination. Tissue samples were fixed in 10% neutral buffered formalin (PBS, pH 7.4). For cryoprotection, the samples were sequentially incubated in 15% and 20% sucrose solutions in PBS. Following sucrose saturation, the specimens were frozen by immersion in isopentane cooled to liquid nitrogen temperature and stored at −80 °C. Frontal sections (15 μm thick) were cut using a Leica CM 1510S cryostat (Leica Microsystems, Wetzlar, Germany). Hippocampal sections corresponding to the stereotaxic coordinates of approximately 3.50 mm caudal to Bregma were selected for analysis according to the rat’s brain atlas [31].

An immunohistochemical analysis was performed to evaluate the distribution of neuronal and microglial markers. The sections were incubated with rabbit polyclonal antibodies against the neuronal nuclear marker NeuN (Fox3, ab177487, Abcam, Cambridge, UK; dilution 1:200), which is selectively expressed in viable neurons, and the microglia marker Iba-1 (ionized calcium-binding adapter molecule 1, K114414P, Solarbio, China; dilution 1:200). For visualization, FITC-conjugated goat anti-rabbit IgG secondary antibodies (ab97050, Abcam, Cambridge, UK; dilution 1:200) were utilized. The specificity of the primary anti-Fox3 antibody (ab177487, Abcam) was ensured by using a commercial, knockout-validated recombinant monoclonal antibody tested by the manufacturer for specific binding in neural tissues. To experimentally verify the specificity of the staining system and rule out non-specific binding of the secondary antibody (ab97050, Abcam), negative controls were routinely performed by omitting the primary antibody. In all negative control sections, no detectable non-specific background fluorescence or background staining was observed. Confocal microscopy was performed using a Leica DMR microscope equipped with a TCS SL laser scanning system (Leica Microsystems, Wetzlar, Germany). The FITC fluorochrome was excited with a 488 nm laser line, and fluorescence emission was recorded within the 496–537 nm range. Cellular composition analysis was carried out using Videotest Master-Morphology software (version 4.0; VideoTest, St. Petersburg, Russia). The numbers of NeuN-positive and Iba-1-positive cells were quantified within 500-μm-wide regions of the CA1 and CA3 hippocampal subfields. NeuN-positive neurons were counted strictly within the stratum pyramidale. Iba-1-positive astrocytes were quantified within an area extending from the stratum oriens to the stratum radiatum. For each animal, final values were obtained by averaging data from 6 individual sections.

### 2.5. Statistical Analysis

Statistical analysis was performed using GraphPad Prism 9 (GraphPad Software, Inc., San Diego, CA, USA). Data distribution was assessed for normality using the Shapiro–Wilk test. Differences in seizure latency and behavioral scores between the HBO_2_ control and various drug treatment groups—including dose-dependent changes and the combination group versus respective monotherapies—were analyzed using one-way ANOVA followed by Tukey’s post hoc test. For histological and morphological quantifications (CA1/CA3 subfields), group differences were evaluated using the non-parametric Kruskal–Wallis test followed by Dunn’s post hoc test for multiple comparisons. Seizure-free survival curves were analyzed using the Kaplan–Meier method and compared via the Log-rank (Mantel–Cox) test. Statistical significance was defined as *p* < 0.05. All data are presented as mean ± standard deviation (SD).

## 3. Results

### 3.1. Characterization of Behavioral Seizure Patterns During HBO_2_ Exposure at 6 ATA

**Figure 1:**
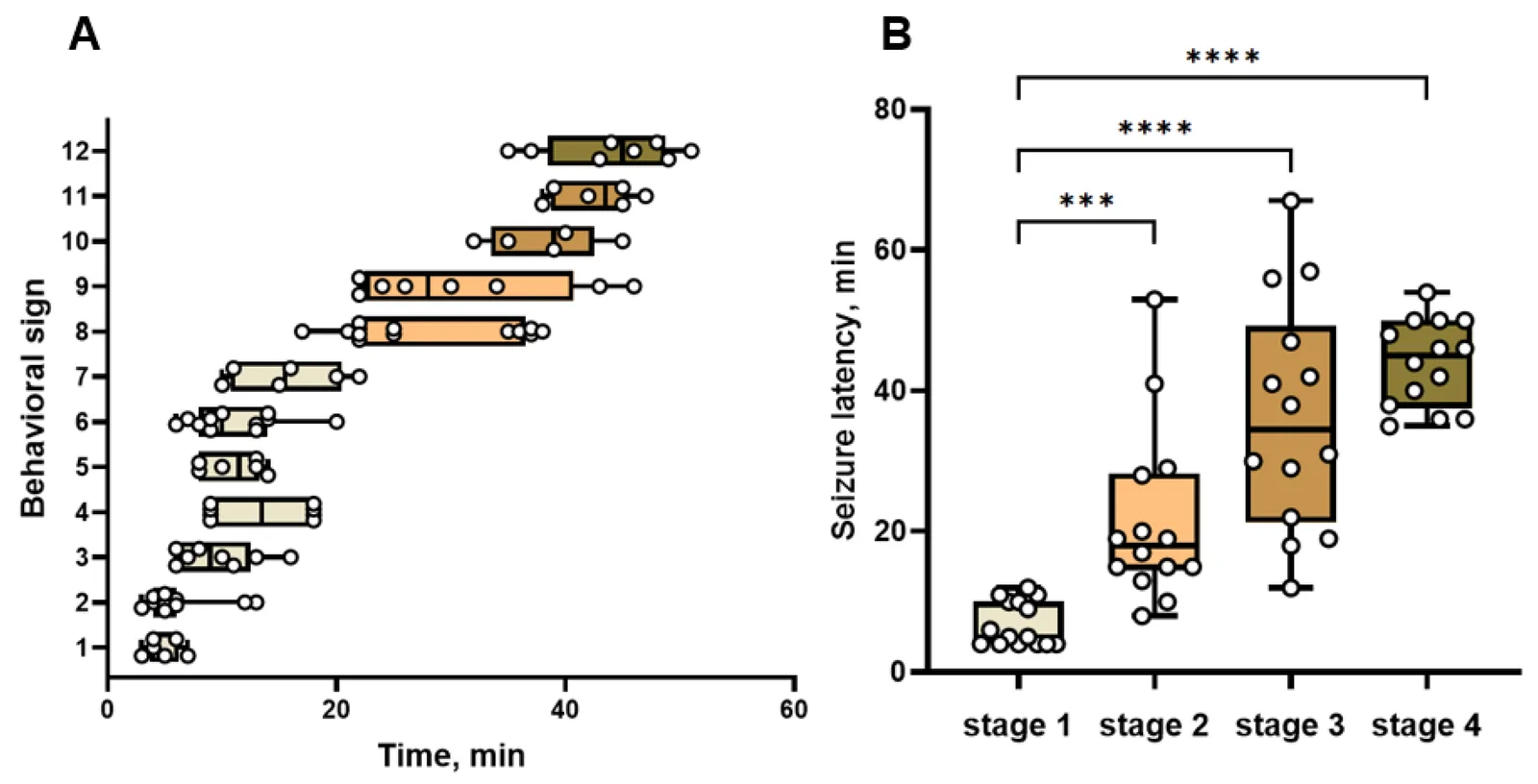
Stage-by-stage development of hyperbaric oxygen-induced seizures in rats at 6 ATA. (**A**) Chronological sequence of the 12 distinct motor disturbances (detailed in the text) relative to oxygen exposure time. (**B**) Latency to the onset of individual seizure stages. Inside each box plot, the central line represents the median, while the lower and upper bounds indicate the 2.5–97.5 percentiles, respectively. The color gradient in panel (**A**) categorizes the behavioral signs according to the stages defined in panel (**B**): signs 1–7 correspond to Stage 1 (light gray), 8–9 to Stage 2 (light brown), 10–11 to Stage 3 (brown), and 12 to Stage 4 (khaki). Asterisks denote statistically significant differences from Stage 1: *** *p* < 0.001, **** *p* < 0.0001.

To evaluate the development of hyperbaric oxygen-induced seizures and systematically classify their progression, we initially performed a detailed analysis of the animals’ baseline behavioral responses. Specific patterns of motor seizure activity were observed in the control group (*n* = 14) during hyperbaric oxygen exposure at 6 ATA. Based on the Racine scale [30], we identified 12 distinct motor disturbances, which were categorized into four stages of seizure severity to track the dynamics of epileptogenesis (Figure 1A). The onset of Stage 1 occurred after the initial 5–7 min of hyperbaric exposure at 6 ATA, during which the animals typically remained motionless. This stage was defined by the appearance of prodromal signs, beginning with intense chewing (1), active licking and grooming (2), brief head shakes (3), and increased locomotor activity (4). These behaviors were followed by isolated facial myoclonus, squinting (5), flattening of the ears (6), and brief (1–3 s) whole-body tremors resembling ‘wet dog shakes’ (7). Stage 2 was characterized by repetitive focal myoclonus of the facial, head, and forelimb muscles (8), frequent whole-body shaking (9) lasting 5–15 s and recurring every few minutes. At Stage 3, the animals developed rhythmic whole-body contractions lasting 10–25 s, often accompanied by rearing (10). This prolonged seizure activity eventually progressed to a loss of balance (falling) and sustained tonic convulsions (11). Stage 4 was defined by generalized tonic–clonic seizures, frequently accompanied by muscle and tail rigidity (12). The latent periods for the onset of Stages 2, 3, and 4 were significantly different from that of Stage 1 (one-way ANOVA, F_(3,53)_ = 33.75, *p* < 0.00001; Tukey’s post hoc test, *p* < 0.001; Figure 1B).

### 3.2. Effect of Methionine Sulfoximine on the Development of HBO_2_-Induced Seizures

**Figure 2:**
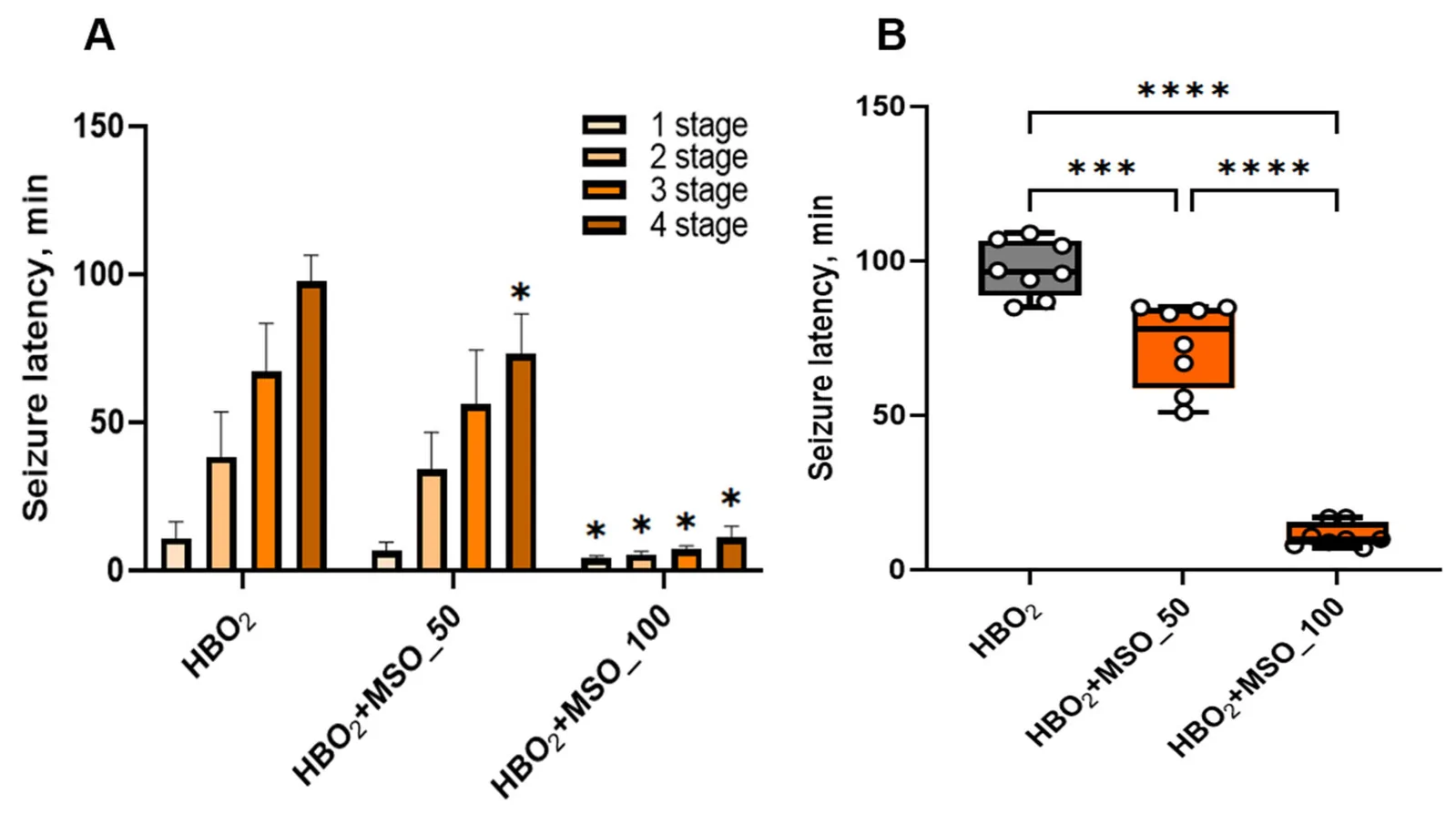
Effect of methionine sulfoximine (MSO) on the development of HBO_2_-induced seizure activity at 5 ATA. (**A**) Latency (min) to the onset of individual stages of seizure severity following the administration of 50 and 100 mg/kg MSO. Data are expressed as mean ± SD. The single asterisk denotes a statistically significant difference relative to control (*p* < 0.05). (**B**) Comparative analysis of latency (min) to Stage 4 generalized seizures between the saline control and MSO-treated groups at 5 ATA. Box plot notations are the same as in Figure 1. In panel (**A**), the single asterisk denotes a statistically significant difference relative to control (*p* < 0.05). In panel (**B**), asterisks indicate highly significant differences in the latency to Stage 4 generalized seizures compared to the saline control group and between MSO doses: *** *p* = 0.0002, **** *p* < 0.0001.

To evaluate the contribution of the glutamatergic system to the mechanisms underlying hyperbaric oxygen seizures, we administered methionine sulfoximine (MSO), a selective glutamine synthetase inhibitor, to increase extracellular glutamate accumulation. Crucially, MSO was utilized as a positive control for glutamate-mediated excitotoxicity to exacerbate seizure development under otherwise submaximal hyperbaric conditions. In this series of experiments, a reduced oxygen pressure of 5 ATA was employed instead of 6 ATA. This adjustment was necessary because higher pressures (6 ATA) triggered generalized seizures too rapidly—often during the compression period—thereby complicating data analysis. By lowering the pressure to 5 ATA, we established a baseline with milder seizure dynamics, providing a suitable experimental window to test whether MSO-induced glutamate accumulation would actively aggravate epileptogenesis, thus confirming the primary role of glutamate in HBO_2_-induced neurotoxicity. Administration of MSO at doses of 50 and 100 mg/kg resulted in a dose-dependent and statistically significant acceleration of the seizure syndrome. Pharmacological blockade of glutamine synthetase markedly reduced the latency to the onset of all stages of seizure severity (one-way ANOVA, *p* < 0.05; Figure 2A). The most pronounced effect of MSO was observed during the analysis of stage 4 generalized seizures (Figure 2B). The 50 mg/kg dose induced a highly significant (*p* < 0.001) decrease in latency, while increasing the dose to 100 mg/kg led to a critical (*p* < 0.0001) reduction in the time to severe paroxysms, shortening the latency by 8-fold compared to the control group (one-way ANOVA, F_(2,21)_ = 168.1, *p* < 0.0001; Tukey’s post hoc test, *p* < 0.0005; Figure 2B).

Taken together, these findings demonstrate that excessive extracellular glutamate accumulation acts as a pivotal trigger that significantly lowers the seizure threshold under hyperbaric hyperoxia. Specifically, the accumulation of endogenous glutamate—driven by the pharmacological inhibition of its conversion to glutamine—drastically accelerates the overexcitation of the central nervous system, thereby establishing a definitive link between disrupted glutamate homeostasis and hyperbaric HBO_2_-induced neurotoxicity.

### 3.3. Paradoxical Acceleration of HBO_2_-Induced Seizures by Memantine

**Figure 3:**
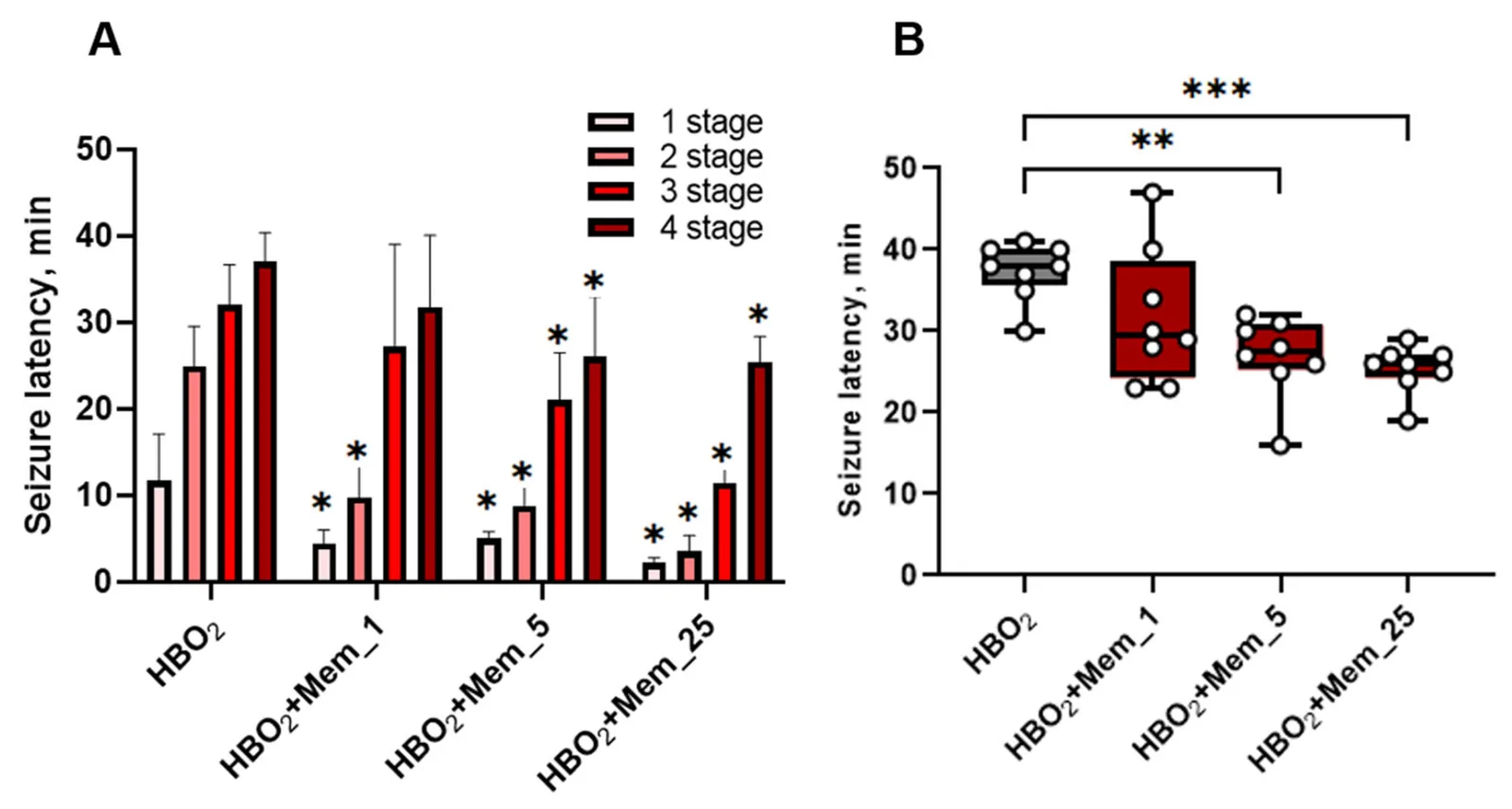
Effect of memantine on seizures induced by HBO_2_ at 6 ATA. (**A**) Latency (min) to the onset of individual stages of seizure severity following the administration of 1, 5 and 25 mg/kg memantine. Data are expressed as mean ± SD. The single asterisk denotes a statistically significant difference between the stages or relative to control (*p* < 0.05). (**B**) Comparative analysis of latency (min) to stage 4 generalized seizures between the control (HBO_2_) and memantine-treated groups. Box plot notations are the same as in Figure 1. In panel (**A**), the single asterisk denotes a statistically significant difference between the stages or relative to control (*p* < 0.05). In panel (**B**), asterisks indicate significant differences in the latency to Stage 4 generalized seizures compared to the saline control group and between memantine doses: ** *p* < 0.01, *** *p* < 0.005.

To determine whether blocking NMDA receptor-mediated excitotoxicity could prevent hyperoxic convulsions, we examined the effects of the non-competitive NMDA receptor antagonist memantine. The staged development of hyperoxic seizures was preserved in all drug-treated animals. However, contrary to the expected neuroprotective outcome, NMDA receptor blockade by memantine significantly accelerated seizure development under hyperbaric oxygen conditions. As illustrated in Figure 3A, memantine (1–25 mg/kg) shortened the latency to the onset of each successive seizure stage (one-way ANOVA, *p* < 0.05). The most pronounced manifestation of this paradoxical response was noted during the analysis of Stage 4 generalized seizures (Figure 3B). Quantitative analysis revealed significant differences between the control HBO_2_ group and the groups treated with 5 and 25 mg/kg of memantine (one-way ANOVA, F_(3,28)_ = 8.123, *p* = 0.0005; Tukey’s post hoc test, *p* < 0.01), whereas the difference between these two specific dosage groups remained statistically non-significant.

### 3.4. Effects of Perampanel on Hyperbaric Oxygen-Induced Seizure

**Figure 4:**
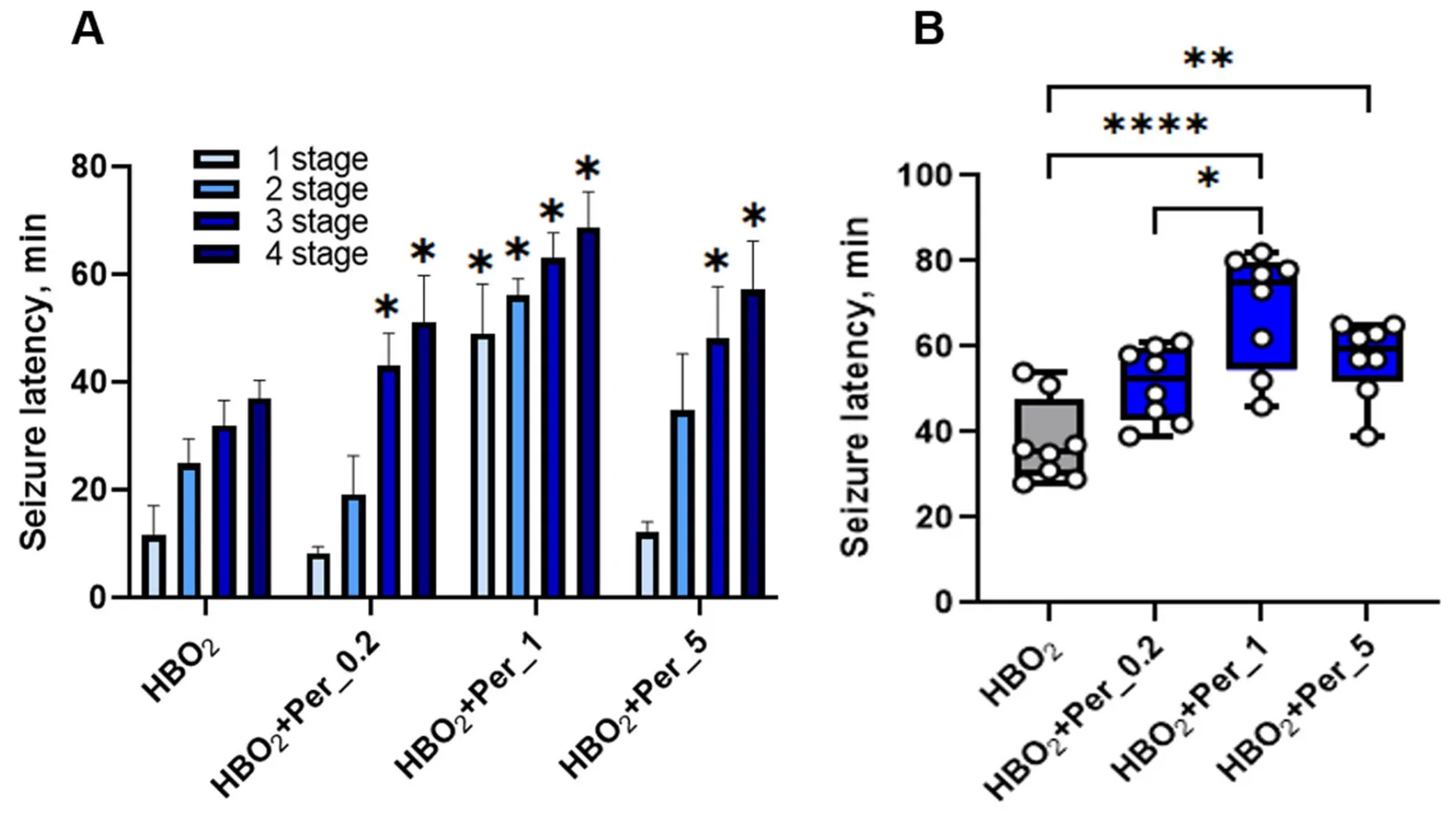
Effects of perampanel on hyperbaric oxygen-induced seizures in rats. (**A**) Latency to individual seizure severity stages. Data are mean ± SD. The single asterisk denotes a statistically significant difference between the stages or relative to control (*p* < 0.05). (**B**) Latency to Stage 4 generalized seizures in control (HBO_2_) vs. perampanel-treated groups (0.2, 1 and 5 mg/kg). Box plot notations are the same as in Figure 1. In panel (**A**), the single asterisk denotes a statistically significant difference between the stages or relative to control (*p* < 0.05). In panel (**B**), asterisks indicate significant differences in the latency to Stage 4 generalized seizures compared to the saline control group and between perampanel doses: * *p* < 0.05, ** *p* < 0.005, **** *p* < 0.0001.

To examine the potential role of ionotropic non-NMDA receptors in oxygen toxicity, we evaluated the effects of perampanel, a selective non-competitive AMPA receptor antagonist. In striking contrast to the NMDA receptor antagonist memantine, pretreatment with perampanel demonstrated a pronounced, dose-dependent protective effect against seizures induced by HBO_2_ at 6 ATA. As illustrated by the latency analysis (Figure 4A), administration of perampanel at doses of 1 and 5 mg/kg significantly prolonged the time to the onset of both Stage 3 and Stage 4 seizures compared to the vehicle control group (one-way ANOVA, *p* < 0.05; Figure 4A). While the lowest tested dose (0.2 mg/kg) exerted no statistically significant effect on seizure initiation, the higher doses (1 and 5 mg/kg) efficiently suppressed the development of generalized tonic–clonic convulsions (Stage 4) and markedly reduced overall seizure severity (one-way ANOVA, F_(3,28)_ = 12.26, *p* < 0.0001; Tukey’s post hoc test, *p* < 0.005; Figure 4B).

Taken together, these results indicate that AMPA receptor activation plays a primary trigger role in the initiation of hyperoxic convulsions. This finding highlights the primacy of AMPA receptors, rather than NMDA receptors, in the mechanisms driving central nervous system oxygen toxicity, representing the most significant mechanistic insight of the present study.

### 3.5. Evaluation of the Combined Administration of Memantine and Perampanel

**Figure 5:**
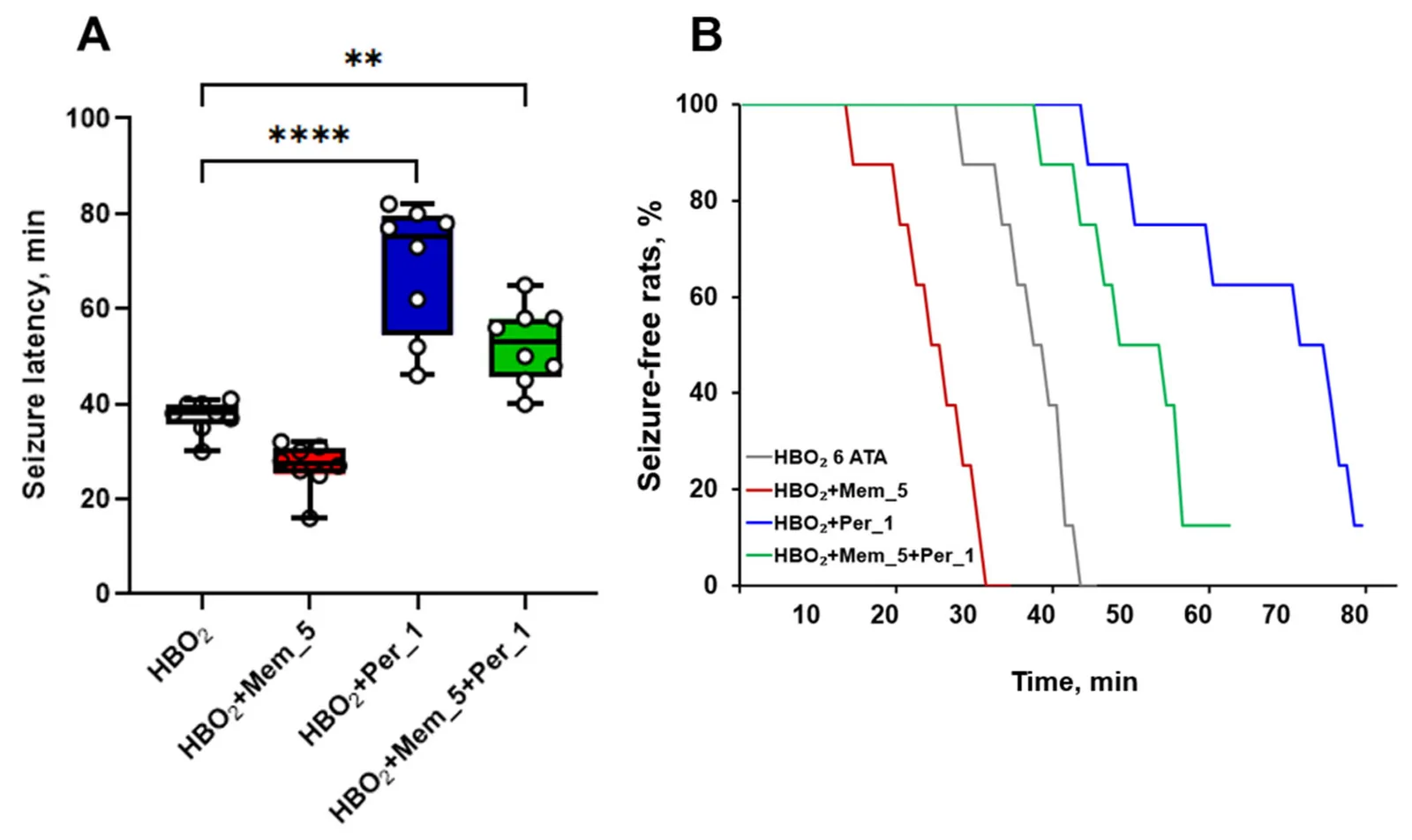
Effects of combined memantine and perampanel administration on hyperbaric oxygen-induced seizures at 6 ATA. (**A**) Latency to Stage 4 seizures in the combination group compared to control and monotherapy animals. For comparative analysis, monotherapy data are replotted from previous experiments shown in Figure 3 (memantine) and Figure 4 (perampanel). Box plot notations are the same as in Figure 1 (one-way ANOVA, ** *p* ˂ 0.01, **** *p* ˂ 0.0001). (**B**) Kaplan–Meier curves demonstrating the percentage of seizure-free animals over time. The combination group shows a significant rescue effect compared to the memantine-only animals (log-rank test, *p* ˂ 0.001).

To determine whether AMPA receptor blockade could counteract the proconvulsant effect of the NMDA receptor antagonist, we evaluated the co-administration of memantine (5 mg/kg) and perampanel (1 mg/kg). For comparative analysis, the baseline data for memantine and perampanel monotherapies were utilized from our previous separate experiments, as detailed in Figure 3 and Figure 4, respectively. Comparative analysis revealed that the anticonvulsant effects of memantine and perampanel differed significantly. The addition of perampanel significantly neutralized the accelerating effect of memantine. In the combined treatment group, the latent period for stage 4 of seizure severity was significantly longer compared to the group receiving memantine alone (one-way ANOVA, F_(3,28)_ = 36.40, *p* < 0.0001; Tukey’s post hoc test, *p* < 0.0001) but did not differ from the group receiving perampanel alone (Figure 5A).

Furthermore, the combined treatment tended to extend the seizure-free interval beyond that of both the saline control and memantine-only groups. Seizures were successfully induced in 100% of the animals in the control HBO_2_ group (6 ATA) and the memantine-treated group. In contrast, protective effects were observed in both the perampanel and the combined memantine + perampanel treatment groups, where seizures developed in only 7 out of 8 rats (87.5%) in each group. Kaplan–Meier analysis confirmed that the combination therapy effectively protected the animals against the rapid onset of generalized seizures observed under memantine monotherapy (log-rank test, *p* < 0.001; Figure 5B).

These findings suggest that AMPA receptor-mediated transmission plays a critical role in the immediate triggering of hyperoxic seizures, and its inhibition can override the paradoxical proconvulsant effects of NMDA receptor blockade.

### 3.6. HBO_2_-Induced Neuronal Loss in the CA3 Region and Its Dependence on NMDA and AMPA Receptor Blockade

**Figure 6:**
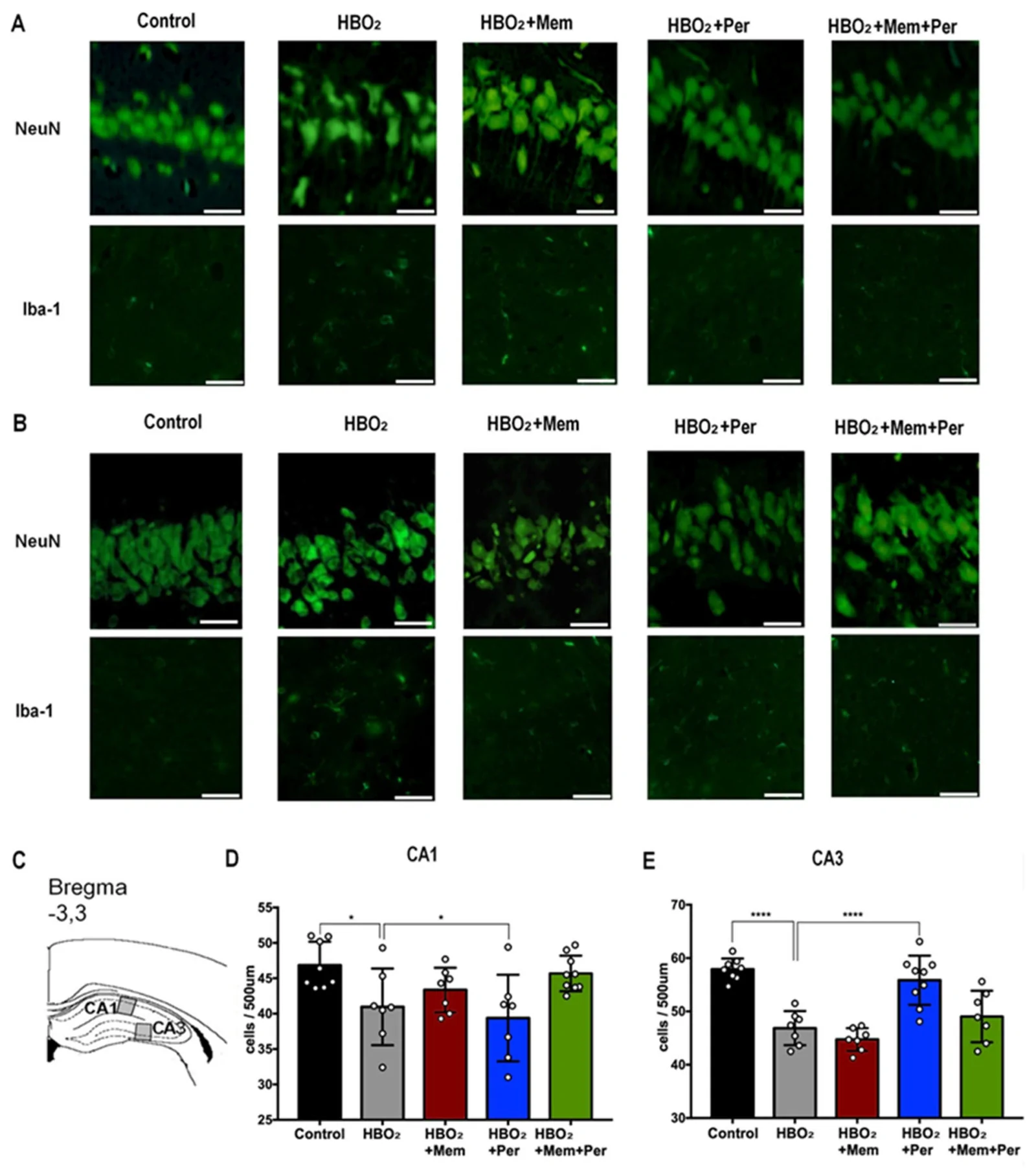
Neuroprotective effects of memantine and perampanel administration in animals exposed to hyperoxic seizures. Hippocampal neurons in rat brains after exposure to HBO_2_ at 6 ATA. (**A**) Photomicrographs of the distribution of NeuN and Iba-1 markers in the pyramidal (NeuN) and radiatum (Iba-1) layers of the CA1 (**A**) and CA3 (**B**). Hippocampal area in rats of the control group, HBO_2_ at 6 ATA rats (HBO_2_ group), and HBO_2_ at 6 ATA rats treated with memantine (HBO_2_ + Mem, 5 mg/kg), perampanel (HBO_2_ + Per, 1 mg/kg), and both memantine and perampanel (HBO_2_ + Mem + Per). Indirect immunofluorescence assay; scale bar is 30 μm. (**C**) Analyzed areas of CA1 and CA3 of the hippocampus. (**D**,**E**) Morphometric analysis of the number of NeuN-positive neurons in areas of CA1 (**D**) and CA3 (**E**) of the hippocampus of rats from the control group (black), HBO_2_ at 6 ATA rats (gray), and HBO_2_ at 6 ATA rats treated with memantine (red), perampanel (blue), and both memantine and perampanel (green). Data are mean ± SD (*n* = 8), * *p* < 0.05 Kruskal–Wallis test with Dunn’s post hoc. **** *p* < 0.0001 one-way ANOVA followed by Tukey’s post hoc test.

This study examined the effects of HBO_2_ on cellular organization and the state of neurons and glial cells in the CA1 and CA3 regions of the dorsal hippocampus. Immunohistochemical staining for the neuronal marker NeuN revealed a significant 19% decrease in the number of viable neurons in the CA3 region of the dorsal hippocampus following HBO_2_ exposure at 6 ATA (one-way ANOVA, F_(4,33)_ = 19.5, *p* < 0.0001; Tukey’s post hoc test, *p* < 0.0001; Figure 6E). In the CA1 region, significant changes in the number of NeuN-positive neurons were also observed (Kruskal–Wallis test, KW = 12.04, df = 4, *p* = 0.02), but the difference between control and HBO_2_ groups was less pronounced (Dunn’s post hoc test: 46.8 ± 1.2 in the control group vs. 40.9 ± 2.0 in the HBO_2_ group) than in CA3. Pre-treatment with memantine (5 mg/kg) prior to HBO_2_ exposure failed to provide a neuroprotective effect in both the CA1 and CA3 regions, as the number of NeuN-positive cells in the HBO_2_ + memantine group did not differ significantly from the HBO_2_-only group (Kruskal–Wallis test, Dunn’s post hoc test, *p* = 0.37 for CA1; one-way ANOVA, Tukey’s post hoc test, *p* = 0.28 for CA3; see Figure 6). Pre-treatment with perampanel (1 mg/kg) prior to HBO_2_ exposure demonstrated a neuroprotective effect in both the CA1 and CA3 regions. Importantly, the number of NeuN-positive cells in the HBO_2_ + perampanel group was significantly higher than in the HBO_2_-only group (Kruskal–Wallis test, Dunn’s post hoc test, *p* = 0.04 for CA1; one-way ANOVA, Tukey’s post hoc test, *p* < 0.0001 for CA3 compared to the HBO_2_-only group; see Figure 6D,E). Pre-treatment with both memantine and perampanel prior to HBO_2_ exposure failed to provide a neuroprotective effect in both the CA1 and CA3 regions (CA1: Kruskal–Wallis test, Dunn’s post hoc test, *p* = 0.74; Figure 6D; CA3: one-way ANOVA, Tukey’s post hoc test, *p* = 0.3). A decrease in the number of viable pyramidal neurons suggested neuronal death in the investigated areas of the hippocampus.

Analysis of Iba-1 immunoreactivity revealed some signs of glial reactivity in response to HBO_2_: some Iba-1-positive microglial cells were observed to be grouped in clusters (Figure 6A,B). Microglial marker (Iba-1) analysis showed that the number of microglial cells in the CA1 and CA3 regions of animals exposed to HBO_2_ (both with and without treatment) did not differ significantly from control levels (one-way ANOVA; CA1: F_(4,33)_ = 1.9, *p* = 0.12; CA3: F_(4,33)_ = 1.5, *p* = 0.22). Thus, the state of microglial cells cannot be fully characterized without additional data on both the levels of proinflammatory cytokines and the phagocytic activity of microglial cells.

## 4. Discussion

Rats exposed to hyperbaric oxygen HBO_2_ at 6 ATA exhibited severe seizure activity characterized by unpredictable muscular contractions ranging from focal myoclonus to generalized convulsions. Although the sequence and timing of onset of individual motor disorders varied across subjects, focal myoclonus of the head, neck, and forelimbs, alongside isolated jerks or whole-body shaking, was consistently observed in every animal prior to the onset of generalized convulsions. These locomotor responses to extreme hyperoxia serve as reliable physiological markers of impending neurotoxicity, the ultimate manifestation of which is generalized tonic–clonic convulsions. These motor disorders align closely with other well-recognized systemic predictors of hyperoxia-induced convulsions in rodents, including increased respiratory rate [32], tachycardia with acute hypertension [33], and altered electrodermal activity [34,35]. Crucially, the presence of these prodromal seizure symptoms points to a progressive, non-linear acceleration of brain activity prior to the generalized fit. In conventional epileptology, such prodromal phases are typically linked to a gradual failure of inhibitory networks; however, under hyperbaric hyperoxia, this pathological hyperactivity is fundamentally driven by an acute, massive enhancement of glutamatergic transmission due to accelerated glutamate accumulation in the synaptic cleft [28].

This specific mechanism is substantiated by our experiments utilizing methionine sulfoximine (MSO) as a metabolic tool. By selectively inhibiting astrocytic glutamine synthetase—the primary enzyme responsible for converting synaptic glutamate into non-toxic glutamine—MSO effectively creates a state of uncontrolled extracellular glutamate bioavailability. While MSO is known to induce slow, delayed seizures over several hours under normobaric conditions in standard epilepsy models due to gradual metabolic exhaustion, its action under HBO_2_ resulted in a dramatic, immediate acceleration of seizure development. This synergistic effect firmly confirms that hyperoxia acts in tandem with disrupted glutamate clearance, demonstrating that overwhelming accumulation of extracellular glutamate and the subsequent hyperactivation of ionotropic glutamate receptors serve as the primary, uncompensated drivers of HBO_2_-induced neurotoxicity.

Further evidence supporting the key role of glutamatergic dysregulation comes from the selective neurodegeneration observed 48 h post-HBO_2_ exposure. We detected a significant decrease in mature NeuN-immunopositive neurons within both the CA1 and CA3 subfields of the dorsal hippocampus, confirming the high vulnerability of this formation to extreme metabolic stress. Pyramidal neurons in these regions feature high mitochondrial density and intense energy metabolism, rendering them highly susceptible to acute oxidative damage induced by hyperbaric hyperoxia [36]. Intriguingly, this pronounced neuronal loss occurred in the complete absence of microglial activation or reactive astrogliosis at the 48 h time point. Under typical traumatic or neuroinflammatory conditions, neural tissue injury triggers rapid glial proliferation and pro-inflammatory signaling [37]. The total lack of glial reactivity observed here suggests a hyper-acute, non-traumatic mechanism of injury. This pattern indicates that the hyperoxic insult either progresses too rapidly for a compensatory glial response to manifest, or directly activates a caspase-dependent intrinsic apoptotic pathway via reactive oxygen and nitrogen species, completely bypassing traditional neuroinflammatory cascades [38,39]. Critically, our analysis revealed that generalized seizure-associated excitotoxicity contributes less to this neuronal death than direct oxidative stress. This is highlighted by the finding that the NMDA receptor antagonist memantine failed to provide any neuroprotection in the CA3 or CA1 fields. In sharp contrast, the non-competitive AMPA receptor antagonist perampanel efficiently prevented hippocampal cell loss. The failure of memantine indicates that hyperoxia-induced neurodegeneration bypasses classical NMDA-mediated excitotoxic pathways. Instead, it points to a distinct mechanism where direct oxidative damage acts in tandem with specific AMPA-mediated pathways, likely driven by the rapid activation of calcium-permeable AMPA channels, which serves as the primary determinant of hippocampal cell death in this model.

Acute oxidative stress drives neuronal damage through both direct RONS-mediated injury and the activation of glutamate receptors that trigger downstream excitotoxicity [40]. Excessive RONS accumulation leads to lipid peroxidation, protein oxidation, and DNA fragmentation, while simultaneously impairing astrocytic glutamate uptake. This failure in clearance causes sustained receptor overstimulation and lethal intracellular calcium (Ca^2+^) overload [41]. Given the selective neuronal death observed without widespread neuroinflammation, caspase-dependent apoptosis directly induced by oxidative stress appears to be the leading degenerative mechanism. While HBO_2_ exposure theoretically engages multiple ionotropic receptor families, our data indicate that NMDA receptor-mediated excitotoxicity contributes negligibly to this neuronal death. Instead, the involvement of calcium-permeable AMPA receptors (CP-AMPARs)—which lack or contain unedited GluA2 subunits and possess high Ca^2+^ permeability—presents a highly plausible pathway for hyperoxia-induced neurodegeneration [41,42,43].

Based on the pivotal role of NMDA receptors in conventional epileptogenesis and our MSO data, we initially hypothesized that the NMDA receptor antagonist memantine would exhibit pronounced anticonvulsant efficacy. Paradoxically, memantine failed to prevent hyperbaric seizures and unexpectedly exacerbated the phenotype, significantly potentiating the proconvulsant impact of hyperoxia. Several distinct mechanisms may account for this phenomenon. First, extreme hydrostatic pressure could directly alter the NMDA receptor macromolecular structure (specifically the GluN2A subunit), preventing effective channel pore blockade by memantine [22,23]. Second, NMDA receptor antagonists might paradoxically enhance glutamatergic neurotransmission by modulating presynaptic release or recruiting alternative binding sites [15,17]. Third, in our opinion, classical NMDA receptors may be functionally bypassed during the initiation phase of hyperbaric seizures. In this scenario, memantine might preferentially block NMDA receptors located on inhibitory GABAergic interneurons, thereby inducing disinhibition of pyramidal networks and accelerating seizure onset under hyperoxic conditions.

The inability of memantine to suppress hyperbaric hyperoxia-induced seizures stands in stark contrast to its well-established anticonvulsant profile across numerous conventional epilepsy models [18,20]. In vitro, memantine efficiently blocks epileptiform activity induced by magnesium-free conditions or direct NMDA application in hippocampal and striatal slices while protecting cultured neurons from glutamate excitotoxicity [18,19,44]. In vivo, systemic administration of memantine (5–20 mg/kg) successfully attenuates acute convulsions induced by chemical convulsants, suppresses audiogenic seizures in Krushinsky–Molodkina rats, and prevents both spontaneous absence-like seizures and cognitive deficits in the lithium-pilocarpine status epilepticus model [45,46,47,48,49,50,51].

However, a closer look at general epileptology literature reveals specific network limitations of NMDA receptor hypofunction that align with our findings. For instance, memantine monotherapy is consistently ineffective against amygdala kindling in rats—a highly refractory model that requires the co-administration of an AMPA receptor antagonist (such as NBQX) to achieve an additive protective effect. Furthermore, while memantine reduces pentylenetetrazole-induced tonic–clonic seizures, dual NMDA/AMPA blockers (such as IEM-1913) exhibit significantly higher anticonvulsant efficacy [52,53].

In our study, the dosages, timing (60 min pre-treatment), and routes of memantine administration strictly corresponded to those validated in vivo protocols, ensuring that NMDA receptors were fully blocked prior to hyperbaric exposure. Therefore, the complete failure of memantine to provide protection—and its paradoxical proconvulsant action—strongly implies that HBO_2_-induced seizures belong to a distinct class of refractory convulsions. These findings lead us to conclude that extreme hyperoxia bypasses classical NMDA-dependent pathways during the initiation phase, shifting the operational priority toward intense, uncompensated AMPA receptor-mediated excitatory neurotransmission.

A second plausible explanation for the lack of anticonvulsant efficacy of memantine involves alterations in cerebral hemodynamics. The development of hyperbaric seizures is highly contingent upon oxygen delivery to the brain, which is directly governed by cerebral blood flow (CBF); an acute increase in CBF significantly shortens seizure latency [25]. Prior studies have demonstrated that MK-801 accelerates HBO_2_-induced seizure onset, potentially because this non-competitive NMDA receptor antagonist induces profound vasodilation and elevates CBF [54]. The resulting enhancement of oxygen delivery to brain tissue exacerbates oxidative stress, accelerating the direct neurotoxic impact of hyperoxia. While this hemodynamic mechanism is well-established for MK-801, whether memantine exerts a similar vasodilatory effect under hyperbaric conditions remains to be fully elucidated.

Alternatively, the failure of memantine may stem from the progressive disruption of GABAergic transmission under extreme hyperoxia. NMDA receptor blockade can paradoxically drive secondary excitation through a network disinhibition mechanism. Because NMDA receptors are critical for maintaining the baseline activity of inhibitory GABAergic interneurons, their inhibition reduces interneuronal output, thereby triggering the disinhibition of cortical and hippocampal pyramidal networks. This hypothesis is supported by the fact that a critical decline in endogenous brain GABA levels is a primary pacemaker of HBO_2_-induced seizures. Mechanistically, hyperoxia-driven RONS directly inhibit glutamate decarboxylase—the enzyme responsible for converting glutamate to GABA—leading to a profound deficit in inhibitory neurotransmission. Under these conditions of depleted GABAergic tone, NMDA receptor blockade is likely insufficient to suppress generalized discharges. Furthermore, extreme hyperoxia specifically potentiates currents through GluN2A-containing NMDA receptors by relieving their tonic inhibition by zinc ions. It is highly plausible that open-channel blockers like memantine exhibit reduced binding efficiency or altered kinetics against this unique, hyperoxia-potentiated mode of receptor activation.

The most significant finding of the present study is the demonstration that AMPA receptor blockade with perampanel substantially attenuates the development of hyperbaric oxygen-induced seizures. While the lowest tested dose (0.2 mg/kg) was ineffective, perampanel at doses of 1 and 5 mg/kg efficiently delayed all stages of seizure severity, ranging from focal myoclonus to generalized tonic–clonic convulsions. Notably, the anticonvulsant efficacy of the perampanel-memantine combination was identical to that of perampanel administered alone. Our Kaplan–Meier survival analysis further indicated that the rapid onset and increased incidence of generalized seizures induced by NMDA receptor blockade can be effectively mitigated by the co-administration of an AMPA receptor antagonist. This evidence confirms a profound functional interaction between these ionotropic glutamate receptor subtypes in the pathogenesis of hyperbaric oxygen toxicity.

As a highly selective, non-competitive AMPA receptor antagonist, perampanel targets the primary drivers of fast synaptic transmission. Due to their rapid gating kinetics and sodium-dependent depolarization, AMPA receptors are uniquely responsible for the initial synchronized depolarization that triggers ictal discharges and drives their propagation across neural networks. By non-competitively preventing glutamate-induced depolarization, perampanel effectively suppresses this synchronized neuronal firing, halting epileptiform propagation even under extreme hyperoxic stress.

In the specific context of central nervous system oxygen toxicity, the complete failure of memantine implies that HBO_2_-induced hyperoxia engages mechanisms that completely bypass or override classical NMDA receptor signaling. While direct hydrostatic pressure or hyperoxia-induced structural modifications—particularly involving the GluN2A subunit—may render NMDA receptors resistant to conventional open-channel blockade by memantine, the resulting network hyperexcitability is fundamentally driven by AMPA receptors. Taken together, our data establish that AMPA receptor activation serves as the definitive upstream trigger for hyperoxic convulsions, identifying these receptors as the primary molecular target for mitigating CNS oxygen toxicity.

While glutamate is the primary neurotransmitter driving synaptic excitation, ionotropic AMPA and NMDA receptors govern distinctly divergent phases of glutamatergic neurotransmission. Structurally and functionally, AMPA receptors possess rapid gating kinetics and sodium-dependent currents that mediate the fast, initial EPSPs necessary to trigger seizure onset (ictal discharge). This rapid activation facilitates the swift propagation of synchronized excitatory signaling across interconnected neural networks. In contrast, classical NMDA receptor recruitment is inherently secondary, acting as a molecular engine for sustaining prolonged seizures (ictal propagation) and downstream excitotoxicity. Under normobaric conditions, the voltage-dependent magnesium (Mg^2+^) block seals the NMDA channel pore; a robust, preliminary AMPA-mediated postsynaptic depolarization is mandatory to expel the (Mg^2+^) ion, thereby allowing sustained calcium (Ca^2+^) influx. Under acute hyperbaric hyperoxia HBO_2_ at 6 ATA, severe oxidative stress and overwhelming accumulation of reactive oxygen and nitrogen species critically alter this relationship, shifting the operational priority of the pathological cascade. The catastrophic rise in extracellular glutamate levels under HBO_2_ exposure creates a hyper-acute state of receptor overstimulation. Because AMPA receptor activation is the upstream gatekeeper for both membrane depolarization and subsequent NMDA channel unblocking, inhibiting this initial phase via non-competitive AMPA antagonism represents the only viable strategy to halt epileptogenesis at its root. By binding to an allosteric site, perampanel effectively neutralizes the synchronized excitotoxic wave before downstream NMDA pathways are even recruited. This foundational bottleneck explains why concurrent NMDA blockade by memantine yields no additional benefit and completely fails to provide neuroprotection in the hippocampus.

Mechanistically, the most critical insight of the present study is the complete reversal of the classic receptor priority typically observed in conventional epileptogenesis. In the vast majority of established experimental seizure models—including pentylenetetrazol, pilocarpine, or kainic acid models—the activation of NMDA receptors serves as the primary molecular trigger that lowers the seizure threshold and drives the transition into status epilepticus. In those standardized setups, NMDA antagonists like memantine routinely exert powerful anti-seizure and neuroprotective effects. Our findings sharply conflict with the established priority of glutamate receptors in epileptogenesis. Under hyperbaric hyperoxia, NMDA blockade not only failed to protect the animals but paradoxically accelerated seizure dynamics, whereas perampanel demonstrated powerful, dose-dependent anticonvulsant efficacy.

Because these data are entirely novel, a direct comparison within hyperbaric physiology is limited; no previous studies have evaluated this specific pharmacological divergence under HBO_2_ exposure. This fundamental contradiction proves that the cascade leading to hyperoxic seizures has a unique etiology.

Unlike standard epilepsy models where slow, high-affinity NMDA currents initiate the seizure, HBO_2_-induced hyperoxia drives a rapid, massive accumulation of synaptic glutamate. This catastrophic neurotransmitter surge likely shifts the network burden onto fast-acting, calcium-permeable AMPA receptors (CP-AMPARs). Under these conditions, conventional NMDA receptor inhibition is ineffective. Furthermore, blocking NMDA receptors under extreme hyperoxia may disrupt remaining network compensation—potentially via the disinhibition of GABAergic interneurons—thereby destabilizing homeostasis and accelerating the onset of convulsions [55,56].

## 5. Conclusions

The present study demonstrates that hyperbaric oxygen-induced seizures and subsequent hippocampal neurodegeneration are primarily driven by early postsynaptic AMPA receptor pathways rather than classical NMDA receptor activation. Our findings establish a critical shift of the conventional receptor priority under extreme hyperoxia, identifying the AMPA receptor as the definitive upstream trigger for hyperoxic convulsions. Pharmacological inhibition with perampanel efficiently mitigates both the behavioral onset of seizures and the selective loss of vulnerable CA3 and CA1 pyramidal neurons, whereas NMDA receptor blockade with memantine fails to provide neuroprotection and paradoxically accelerates seizure dynamics. Collectively, these results reveal a unique, hyper-acute pathophysiological etiology for central nervous system oxygen toxicity, shifting the fundamental focus of hyperbaric physiology toward fast-acting ionotropic glutamate receptors as the primary molecular targets for future protective intervention.

## Data Availability

The original contributions presented in this study are included in the article. Further inquiries can be directed to the corresponding author.
